# How Endothelial Cells Adapt Their Metabolism to Form Vessels in Tumors

**DOI:** 10.3389/fimmu.2017.01750

**Published:** 2017-12-11

**Authors:** Annalisa Zecchin, Joanna Kalucka, Charlotte Dubois, Peter Carmeliet

**Affiliations:** ^1^Laboratory of Angiogenesis and Vascular Metabolism, Vesalius Research Center, VIB, Leuven, Belgium; ^2^Laboratory of Angiogenesis and Vascular Metabolism, Department of Oncology, KU Leuven, Leuven, Belgium

**Keywords:** angiogenesis, metabolism, sprouting, tip and stalk cells, tumor angiogenesis

## Abstract

Endothelial cells (ECs) line blood vessels, i.e., vital conduits for oxygen and nutrient delivery to distant tissues. While mostly present as quiescent “phalanx” cells throughout adult life, ECs can rapidly switch to a migratory “tip” cell and a proliferative “stalk” cell, and sprout into avascular tissue to form new blood vessels. The angiogenic switch has long been considered to be primarily orchestrated by the activity of angiogenic molecules. However, recent evidence illustrates an instrumental role of cellular metabolism in vessel sprouting, whereby ECs require specific metabolic adaptations to grow. Here, we overview the emerging picture that tip, stalk, and phalanx cells have distinct metabolic signatures and discuss how these signatures can become deregulated in pathological conditions, such as in cancer.

## The Basics of Endothelial Cell (EC) Biology

**Figure 1:**
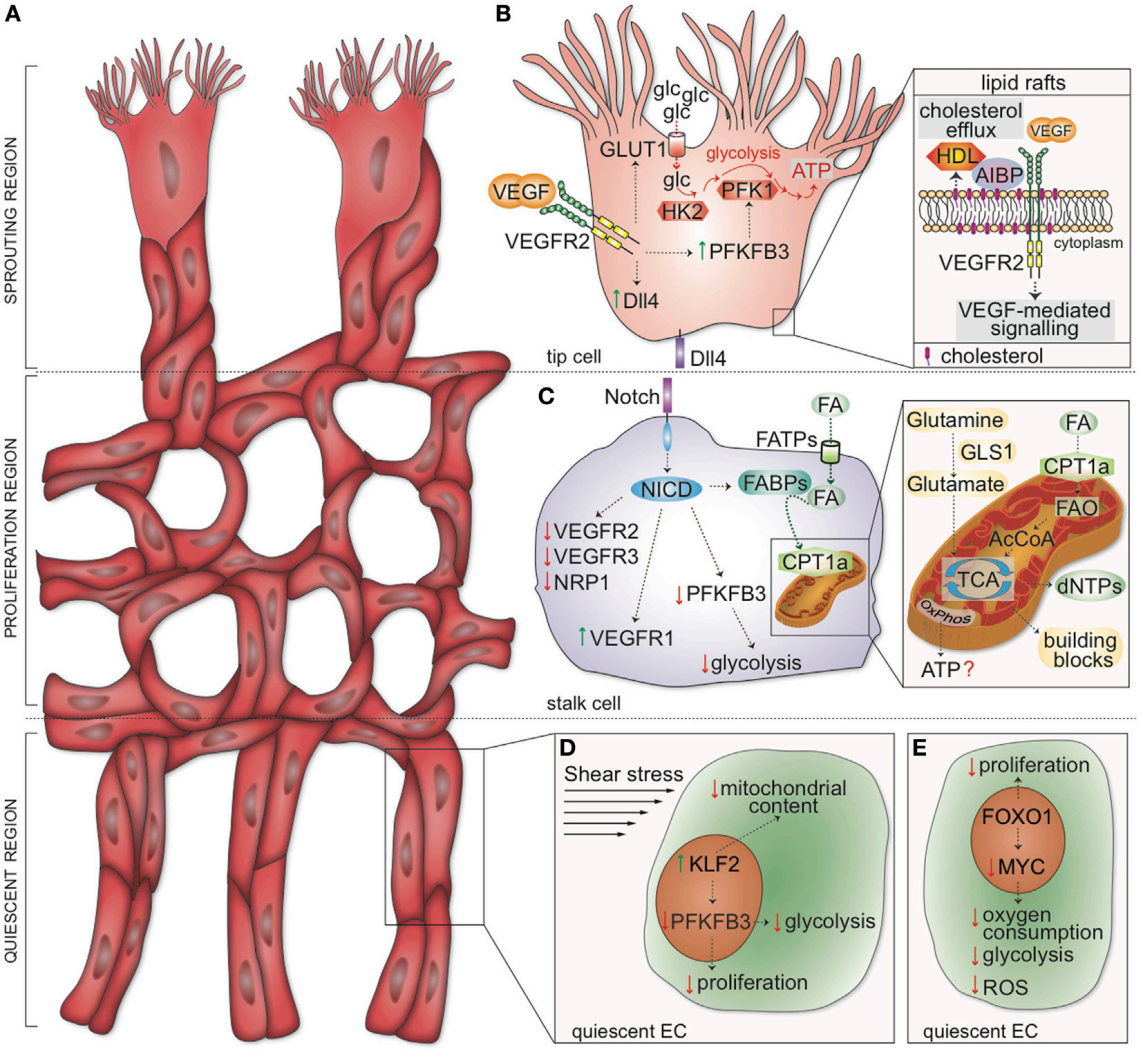
Metabolic and genetic determinants regulating tip, stalk, and phalanx cell function. **(A)** Schematic representation of the vascular front. Three distinct zones are identified: the sprouting region (consisting of migrating tip cells), the proliferation region (containing stalk cells elongating the sprouts), and the quiescent region (where phalanx cells mature into quiescent cells). **(B)** In tip cells, VEGF-mediated activation of VEGFR2 induces transcription of Dll4. Concomitantly, VEGFR2 signaling upregulates GLUT1 and PFKFB3, thus increasing glucose uptake and glycolysis to induce localized adenosine triphosphate (ATP) generation at lamellipodia and filopodia. HK2 is also required for tip cell functions. Inset: cholesterol transfer to HDL is facilitated by AIBP; the rate of cholesterol efflux from the plasma membrane determines the formation of lipid rafts, positively affecting VEGFR2 dimerization and activation. **(C)** In stalk cells, Dll4 induces the cleavage of the NICD. In turn, NICD drives the expression of VEGFR1 while downregulating VEGFR2, VEGFR3, and NRP1. FAs are taken up into the cells by FATPs and, in the cytoplasm, bound by FABP. NICD represses PFKFB3 transcription and enhances FABP expression. Inset: CPT1a shuttles FAs into the mitochondria. Mitochondrial FAO generates acetyl-CoA, which enters the TCA cycle and supports dNTP synthesis; OxPhos is seemingly used for minimal ATP generation in endothelial cells (ECs). The conversion of glutamine to glutamate by GLS1 sustains the TCA cycle (anaplerosis) and contributes to building block production. **(D)** Shear stress induces the expression of KLF2, which in turn inhibits proliferation, reduces the mitochondrial content, and lowers glycolysis (by downregulating PFKFB3). **(E)** FOXO1 suppresses EC proliferation and inhibits the transcription factor MYC. As a consequence, oxygen consumption, glycolysis, and reactive oxygen species (ROS) production are lowered in quiescent ECs. VEGF, vascular endothelial growth factor; VEGFR2, vascular endothelial growth factor receptor 2; glc, glucose; GLUT1, glucose transporter-1; PFK-1, phosphofructokinase; PFKFB3, 6-phosphofructo-2-kinase/fructose-2,6-bisphosphatase 3; ATP, adenosine triphosphate; HK2, hexokinase 2; AIBP, apoA-I binding protein; Dll4, Delta-like ligand 4; HDL, high-density lipoprotein particles; NICD, Notch intracellular domain; FA, fatty acids; FATP, FA transporter protein; FABP, FA binding proteins; CPT1a, carnitine palmitoyltransferase 1a; FAO, FA β-oxidation; AcCoA, acetyl coenzyme A; TCA tricarboxylic acid cycle; OxPhos, oxidative phosphorylation; dNTPs, deoxynucleotides; GLS1, glutaminase 1; FOXO1, forkhead box O transcription factor 1; KLF2, Krüppel-like Factor 2.

Endothelial cells line blood vessels, an intricate network of functional conduits throughout the body, which is vital for the maintenance of tissue homeostasis. In healthy adults, ECs remain in a quiescent state for protracted periods. Nevertheless, quiescent blood vessels retain the ability to promptly respond to pro-angiogenic cues in the tightly coordinated process of angiogenesis. The angiogenic switch is characterized by active proliferation and migration of ECs to form new sprouts. A key player that orchestrates vascularization of oxygen- and nutrient-deprived tissues is vascular endothelial growth factor A (VEGF-A, hereafter referred to as VEGF), which signals primarily through VEGF receptor 2 (VEGFR2) (1). For vessel branching to occur, activated ECs must differentiate into three different subtypes, namely (i) migratory tip cells, which lead the sprout, (ii) proliferating stalk cells, responsible for sprout elongation, and (iii) quiescent phalanx cells, which line the newly established perfused vessel (1) (Figure 1A). The distinct morphological features and functional properties that distinguish these EC subtypes will be summarized in the next section. The angiogenic switch is metabolically taxing; recent evidence suggests that ECs adapt their metabolism during vessel sprouting to fulfill their needs of biomass production and to sustain high-energy requirements (2, 3). Furthermore, EC metabolism has been proven to co-determine vessel sprouting; thus, challenging the broadly accepted belief that angiogenesis is mainly (only) governed by angiogenic (growth factor) signals (2).

Here, we will briefly overview some key angiogenic determinants of vessel sprouting and EC quiescence [for further details, the reader is referred to more comprehensive reviews (1, 4, 5)]. We will then discuss metabolic pathways that are activated in angiogenic versus quiescent ECs, with particular emphasis to the metabolic reprogramming underlying the angiogenic switch.

## Genetic Signals in the Regulation of Vessel Sprouting

The primary function of tip cells is to lead the developing sprout in response to pro-angiogenic cues. Tip cells have a minimal proliferation rate and are characterized by a particular morphology: they are highly polarized and extend numerous filopodia and lamellipodia to allow cellular movements (6). Moreover, tip cells are rich in cell-surface receptors and molecules involved in extracellular matrix degradation and basement membrane deposition (7). On the contrary, stalk cells generate fewer filopodia and proliferate behind the tip cells to ensure sprout elongation and lumen formation (8). The specification of ECs into tip and stalk cells is tightly orchestrated by the VEGF and Notch signaling pathways (5). At the vascular front, the ECs exposed to the highest concentration of VEGF are selected to become tip cells. Activation of the cognate receptor VEGFR2 initiates an intracellular cascade in tip cells that induces the expression of the Notch ligand delta-like ligand 4 (Dll4) (5) (Figure 1B). Subsequent activation of Notch signaling in neighboring ECs *via* Dll4 binding results in the cleavage of the Notch intracellular domain (NICD); in turn, the NICD activates a transcriptional program that results in reduced expression of VEGFR2, VEGFR3, and the VEGF coreceptor neuropilin 1 (NRP1), with concomitant upregulation of the VEGF trap VEGFR1 (5) (Figure 1C). Thus, Notch signaling out-competes the ability of an EC to become a tip cell and instead promotes a stalk cell phenotype. Moreover, during angiogenic sprouting, the high turnover of VE-cadherin, a key junctional adhesion molecule in ECs, facilitates cell migration (9). Notch signaling also reduces the adhesiveness of VE-cadherin junctions and consequently compromises the ability of these cells to reshuffle positions within the sprout (10). However, the phenotype of tip and stalk cells is not fixed: dynamic rearrangements occur as the vessel network expands; with tip cells frequently being overtaken by stalk cells that move to the front and become new tips (11, 12). Notably, a novel EC topology was recently described in growing vessels, according to which at least two filopodia-extending ECs are present at the tip of the sprout (13). The polarization of tip ECs along the longitudinal border seemingly allows for apical polarization and lumen formation (13).

The process of anastomosis, the contact between tip cells of neighboring sprouts, establishes new vessel connections (1). Once tissue vascularization is restored, the levels of pro-angiogenic factors are reduced and ECs establish a quiescent phenotype. Quiescent ECs, also termed phalanx cells, are highly interconnected by junctional molecules such as VE-cadherin and tight junction proteins that mechanically strengthen the vessel’s wall and create a barrier (1). Perfusion induces vascular maturation by re-establishing pericyte recruitment and maturation and basal membrane deposition to promote vessel stabilization (1).

## Metabolism at the Tip: Glycolysis as a Fuel

Endothelial cells are exposed to high concentrations of oxygen in the blood. Oxidative phosphorylation would, therefore, be expected to be the favored energy-generating pathway. On the contrary, only <15% of the total amount of ATP is produced through oxidation of glucose, glutamine, and fatty acids (FA), and sprouting ECs have a lower oxygen consumption rate than other cell types (2). In turn, glycolysis yields 85% of the total cellular ATP content (2). Even if it seems counterintuitive at first sight, as the yield of ATP per mole glucose from mitochondrial respiration (36 mol ATP) is much higher than from anaerobic metabolism (2 mol ATP), the preference for glycolysis might have several advantages. First, in conditions of unlimited glucose, high glycolytic flux can produce more ATP in a shorter time with respect to oxidative metabolism (14), which represents an advantage for cells that have to rapidly migrate into avascular tissues in order to restore the physiological oxygen levels. Also, by relying primarily on anaerobic metabolism for ATP production, ECs spare oxygen for transfer to perivascular cells. In addition, glucose can also be shunted into glycolytic side pathways such as the pentose-phosphate pathway and the serine biosynthesis pathway utilized for biomass production (15). Last, a reduction in mitochondrial respiration reduces the amount of reactive oxygen species (ROS) generated (16).

Upon VEGF stimulation, ECs rewire their metabolism to meet the elevated energetic and biosynthetic needs of the angiogenic state. The VEGF-dependent upregulation of the glycolysis regulator 6-phosphofructo-2-kinase/fructose-2,6-bisphosphatase 3 (PFKFB3), an activator of phosphofructokinase 1, is paralleled by an increase in glycolytic flux (2) (Figure 1B). Notably, activation of the pro-stalk signaling Notch receptor *via* Dll4 reduces PFKFB3-driven glycolysis (Figure 1C), arguing for a high glycolytic activity as metabolic feature of tip cells (2). Consistently, genetic loss or pharmacologic inhibition of PFKFB3 diminishes tip cell behavior and reduces competitiveness for the tip position, consequently impairing EC sprouting *in vitro* and vessel outgrowth *in vivo* (2). On the contrary, overexpression of PFKFB3 reverts Notch-instructed stalk cells into tip cells, suggesting that glycolysis can even override genetic signals modulating EC specification (2). Besides PFKFB3, the glycolytic enzyme hexokinase 2 (HK2) has been recently implicated in the processes of angiogenesis and lymphangiogenesis. Pan-endothelial *Hk2* deletion during embryonic development induces defects in angiogenesis, and *Hk2* excision soon after birth (P0) impairs the development of the retinal vasculature by reducing the number of both tip cells and branch points (17).

In motile ECs, bulky mitochondria are positioned in the perinuclear cytosol; glycolytic enzymes, instead, associate with F-actin and compartmentalize in lamellipodia and filopodia (2) to generate high and localized amounts of ATP. Thus, glycolysis fuels actin–myosin contraction and enables cytoskeleton remodeling during EC migration by locally producing high amounts of ATP (2). Dynamic rearrangements at the tip of the sprout allow for a continuous selection and repositioning of the more competitive tip cell to ensure maximal fitness of the growing sprout (11, 12). Remarkably, computational simulations, validated by experimentations, predicted that glycolytic ATP generation, required for cellular rearrangements, modulates intercellular adhesion by affecting VE-cadherin turnover (18). Of note, these computational simulations predicted that PFKFB3 blockade in combination with anti-VEGF treatment might normalize vessel sprouting in models of deregulated EC rearrangement due to excessive VEGF levels (as observed in conditions of pathological angiogenesis) (18). Lowering glycolysis in ECs by genetic and/or pharmacological means indeed promotes tumor vessel normalization (19).

Glycolysis is not the only metabolic determinant of tip cells. Indeed, cholesterol turnover and efflux are indispensable to maintain normal cellular functions. Generally, cholesterol is loaded onto ApoA-1-containing high-density lipoprotein particles (HDL) in an ATP-binding cassette transporters-dependent fashion (20). The apoA-I binding protein (AIBP) favors cholesterol transfer from ECs to HDL (Figure 1B). In the presence of AIBP and HDL, the rate of cholesterol efflux is accelerated, thereby impairing formation of plasma membrane lipid rafts, essential for VEGFR2 dimerization and endocytosis, overall thus inhibiting VEGF-mediated signaling (20). In zebrafish embryos, tip cells have a higher content of lipid rafts than stalk cells, suggesting a cholesterol-dependent positive regulation of VEGFR2 signaling at the tip of the sprout (20). Interfering with cholesterol efflux abrogates these morphological differences and results in dysregulated angiogenesis (20).

Furthermore, glutamine deprivation or inhibition of glutaminase 1 (GLS1), an enzyme of amino acid metabolism that converts glutamine to glutamate, negatively affects tricarboxylic acid (TCA) cycle anaplerosis, macromolecule production, and redox homeostasis in ECs (21, 22). The inhibition of glutamine metabolism impairs EC proliferation and migration, causes severe vessel sprouting defects *in vivo*, and decreases pathological ocular angiogenesis (21, 22). In particular, the silencing of GLS1 in ECs reduces their competitiveness to obtain the tip position in spheroid sprouts *in vitro* (21). In a separate study, microarray analysis of ECs dissected from the postnatal retinal vasculature showed increased expression of glutaminase 2 (GLS2) (23). Yet, the exact role of GLS2 in angiogenesis is still unclear.

## Metabolic Determinants of Stalk Cell Proliferation

Despite the pivotal role of glycolysis in the specification and functioning of tip cells, this metabolic pathway is also associated with stalk cell functions. Indeed, these actively proliferating cells need to generate high amounts of biomass (macromolecules) that are in part derived from the non-oxidative pentose-phosphate pathway and the serine biosynthesis pathway, side branches of glycolysis (24). Rapidly proliferating ECs, such as tumor ECs, increase glucose uptake, diversion of glycolytic intermediates into these anabolic pathways, and incorporation of glucose carbons into nucleotides (19). Consistently, treatment of ECs with Dll4 (a Notch signaling activator) suppresses progression through the cell cycle while concomitantly reducing glycolysis at the late G_1_ cell cycle phase (25). In addition, PFKFB3 inhibition *in vitro* impairs EC sprouting induced by Notch blockade (2), thus underscoring the ability of the glycolytic activator PFKFB3 to overcome the metabolic break induced by Notch.

Fatty acid metabolism is also essential for EC proliferation (Figure 1C). The uptake of FAs across the plasma membrane can occur *via* a “flip-flop” mechanism or *via* specific transport proteins, such as CD36 and FA transport proteins (26). Once shuttled to the inner side of the plasma membrane, FAs are recruited by FA binding proteins (FABPs), which are responsible for their intracellular trafficking (26). Of note, FABP4 regulates proliferation and sprouting of ECs (27, 28). The shuttling of activated FA-CoA to mitochondria is dependent on the activity of the carnitine palmitoyltransferases (CPTs), a rate-controlling step of FA β-oxidation (FAO) (29). Genetic or pharmacological inhibition of CPT1a, the most abundant isoform in ECs, reduces EC proliferation without affecting the migratory capability of ECs (30). Endothelial CPT1a loss in mice impairs postnatal vascular development of the retina and reduces branching angiogenesis without altering filopodia formation or vessel maturation, thus suggesting that FAO exquisitely modulates stalk cell behavior (30). In addition, pharmacological inhibition of FAO affects the barrier function by inducing EC hyper-permeability (31).

A role for oxidative phosphorylation in ATP generation was documented in proliferating ECs (32) (Figure 1C). However, the magnitude and relevance of these findings remain debated, since oxidative metabolism in ECs accounts for the generation of only less than 15% of ATP (2) and, consistently, the defect observed upon CPT1a silencing is not due to insufficient ATP production or defective oxygen consumption (30). Instead, ^13^C-palmitate tracing experiments uncovered that FAO generates acetyl coenzyme A (acetyl-CoA), which helps to sustain, in conjunction with an anaplerotic substrate, the TCA cycle and deoxynucleotide (dNTP) synthesis for proliferation (30) (Figure 1C). In line with these observations, silencing of CPT1a depletes the cellular pool of dNTPs, which are restored upon supplementation with acetate, a precursor of acetyl-CoA (product of FAO) (30). Interestingly, acetate- or dNTP supplementation recovers the sprouting defect observed upon CPT1a silencing *in vitro* (30). Thus, the selective role of FAO in stalk but not tip cells implies distinct metabolic signatures for these different EC subphenotypes.

## Metabolism of Quiescent Phalanx ECs

When cells are exposed to unfavorable conditions to sustain cell proliferation, many healthy (non-transformed) cells enter a non-dividing state while still keeping the ability to become proliferative. This state of reversible cell cycle arrest in the G_0_/G_1_ phase, also known as quiescence, is common in many non-transformed cell types in different species (e.g., bacterial, yeast, or mammalian cells). The switch from quiescence to proliferation (and *vice versa*) is accompanied with substantial changes in metabolism. As an example, quiescent T cells derive most of their ATP from oxidative phosphorylation (33, 34). By contrast, activated T cells rely on glycolysis, facilitated by increased glucose transport (35). Similarly, B-cell activation in response to interleukin-3 induces a 8-fold increase in glycolytic flux (36).

As mentioned above, ECs build new blood vessels when oxygen and nutrients are low and become quiescent once functional vessels have been perfused, and levels of oxygen and nutrients are restored (1, 4). Although the metabolism of quiescent ECs remains poorly characterized, recent data showed that ECs rewire their metabolism when exiting the cell cycle. Upon (genetic or chemical) inhibition of PFKFB3, proliferating ECs lower their glycolytic flux by 35–40% and display a quiescent phenotype (2, 37, 38). Similar results have been obtained upon Kruppel-like factor 2 (KLF2) overexpression, which mimics the anti-proliferative effect induced by laminar shear stress. KLF2 overexpression, as well as shear stress itself, reduces mitochondrial content and activity and causes a decrease in glycolytic flux to the same levels observed upon PFKFB3 inhibition (39). Mechanistically, KLF2 represses the transcription of PFKFB3 (39) (Figure 1D). Of note, overexpression of PFKFB3 in ECs restores glycolytic flux and overcomes KLF2-mediated inhibition of proliferation (39).

Another recent study highlighted a role for forkhead box O (FOXO) transcription factor 1 (FOXO1) in coupling cellular growth/quiescence to metabolism in ECs (Figure 1E). Expression of constitutively active FOXO1 suppresses EC proliferation and lowers endothelial metabolic activity by decreasing glycolysis, lactate production, oxygen consumption, and the production of ROS (38). These changes in metabolic activity are promoted by FOXO1’s ability to antagonize MYC signaling (38).

In a healthy adult mammalian organism, the quiescent endothelium has a long half-life of several years, and as long as it remains healthy, it secures vascular homeostasis through vasculoprotective mechanisms, including vasodilation, thromboresistance, anti-inflammation, etc. (40). However, upon aging, quiescent ECs risk becoming dysfunctional, thereby contributing to the progression of various cardiovascular diseases (CVD). The metabolic features of dysfunctional ECs during aging have not been fully elucidated yet and further studies are warranted to shed light on the mechanisms of metabolic (mal)-adaptation, but because of the importance of EC dysfunction in CVD, we will briefly discuss the current knowledge on this topic.

## Aging and Metabolism in ECs

Advanced age is an independent risk factor for life-threatening diseases, including coronary artery disease, stroke, and hypertension, which are directly related to aging-associated EC dysfunction (41). With time, aging blood vessels become stiffer and thicker, which results in decreased flexibility in adjusting vessel shape and function in response to tissue demands. The age-associated remodeling of the vasculature involves an impairment of vasorelaxation, an increase in vascular permeability, inflammation, and fibrosis, and an impairment of angiogenesis (42–44).

Emerging evidence arises about the underlying (metabolic) mechanisms of the aging-associated EC dysfunction. A common mechanism seems to be mitochondrial DNA damage and mitochondrial dysfunction, with decreased production of ATP and upregulation of ROS production (45, 46). The mitochondrial respiration chain and NAD(P)H oxidases (Nox) are important sources of ROS production in ECs (47). In aged animals, inflammatory markers such as the intercellular adhesion molecule 1 (ICAM1), a leukocyte adhesion molecule that promotes vessel wall inflammation, are upregulated with respect to young animals (48). This increased expression appears to be partially dependent on ROS, as long-term treatment with mitochondria-targeted antioxidant treatment abrogates the increased expression of ICAM1 (48). Also, the NADPH Oxidase 4 (Nox4), which is vasculoprotective at a young age (49), is upregulated in pulmonary arteries of aged rats and has been implicated in oxidative stress (50). In agreement, upon knockdown of Nox4, the replicative lifespan of ECs is extended and oxidative DNA damage is reduced (51). In addition, a reduction in Sirtuin 1 activity in ECs has been proposed to contribute to vascular dysfunction in aging mice by reducing FA uptake and oxidation, and by inducing mitochondrial dysfunction, oxidative stress, and DNA damage (52). Not only the production of ROS is increased, but also ROS scavenging is impaired in the aged endothelium (50).

Endothelial cell dysfunction during vascular aging is also associated with reduced endothelial nitric oxide (NO) bioavailability. NO is an important cellular signaling molecule that mediates vasodilatation and stimulates angiogenesis (53). However, aging-induced oxidative stress can change the fate of endothelial NO synthase (eNOS) from a NO-producing to a superoxide anion-producing enzyme (in a process known as eNOS uncoupling), resulting in impaired NO generation (54).

Aging dysfunctional ECs may, thus, create a milieu in which vascular disease can flourish. It is, therefore, of great clinical relevance to deepen our knowledge on the mechanisms underlying age-related EC dysfunction and to assess benefits of interventions that may restore EC function.

## Metabolism of ECs in the Tumor Microenvironment

**Figure 2:**
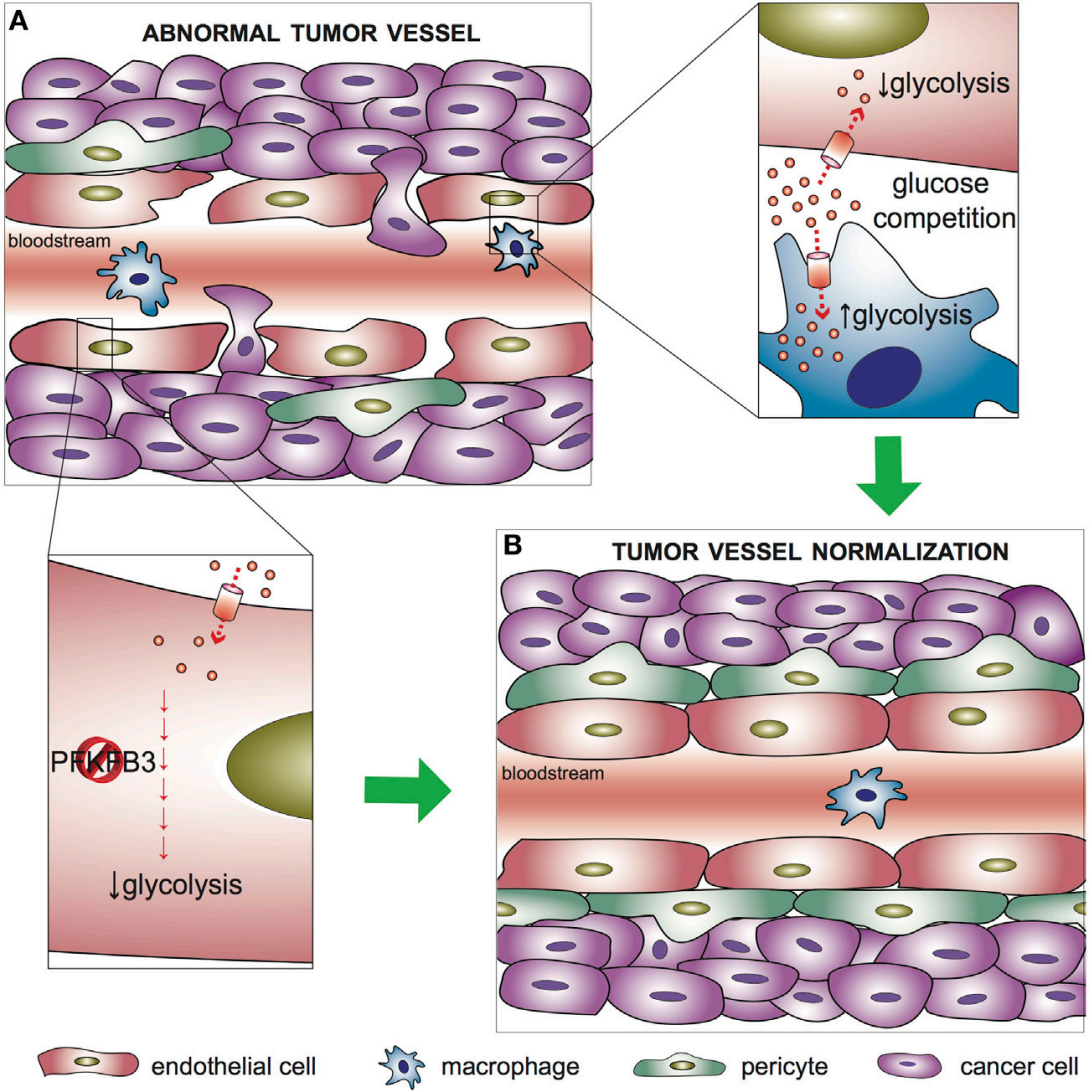
Metabolic regulation of tumor vessel normalization. **(A)** In abnormal tumor vessels, endothelial cells (ECs) form weak junctions that, in combination with reduced pericyte coverage, result in increased leakiness and facilitate intravasation of cancer cells. **(B)** Targeting glycolysis in tumor ECs, which are hyperglycolytic with respect to healthy ECs, promotes normalization of the tumor vasculature. Upper left inset: tumor-associated macrophages (TAMs) compete with ECs for glucose availability in the tumor microenvironment; an increased glycolytic flux in TAMs favor tumor vessel normalization. Lower left inset: targeting PFKFB3 to partially reduce glycolysis in ECs improves vessel maturation, favors the tightening of the vascular barrier, and reduces cancer cell intravasation. PFKFB3, 6-phosphofructo-2-kinase/fructose-2,6-bisphosphatase 3.

The tumor microenvironment is characterized by a highly disorganized vessel network, which is morphologically and functionally abnormal. The vasculature appears irregular, tortuous, and non-homogeneous, and the ECs form weak junctions that result in increased leakiness and facilitate intravasation of cancer cells (4). A recent study demonstrated that the metabolic profile of ECs lining tumor blood vessels differs from healthy ECs (19). In particular, most glycolytic genes, including PFKFB3, are upregulated at the transcriptional level with respect to other pathways in central metabolism, thus supporting a higher dependence of tumor ECs on glucose metabolism (19). In agreement with these findings, competition for the availability of glucose between tumor-associated macrophages (TAMs) and tumor ECs influence the angiogenic response. Indeed, enhancing glycolysis in TAMs reduces glucose availability for ECs in the tumor microenvironment and favors normalization of the tumor vasculature (55) (Figure 2). In mice, endothelial deletion of both PFKFB3 alleles results in reduced vessel perfusion in tumors (56). Consistently, treatment with a high dose of the PFKFB3 blocker 3-(3-pyridinyl)-1-(4-pyridinyl)-2-propen-1-one (3PO) suppresses tumor EC proliferation and promotes EC death, ultimately causing tumor vessel disintegration (57). Surprisingly, however, PFKFB3 haplodeficiency or the treatment with a low dose of 3PO, which target the hyperglycolytic tumor ECs (but not the cancer cells), do not affect tumor growth but instead exert a favorable effect on tumor vessel normalization, characterized by remodeling of the tumor vasculature into a more normal one, with a tighter vascular barrier, more pericyte coverage (vessel maturation), and improved vessel perfusion and oxygenation. This results in reduced metastasis and amelioration of the delivery and response to chemotherapeutic agents (19) (Figure 2). A growing body of evidence in the past years has highlighted the efficacy of tumor vessel normalization in improving tumor perfusion and oxygenation as well as anti-cancer drug delivery (58). The finding that partial inhibition of glycolysis induces tumor vessel normalization paves the way for further investigations on the modulation of EC cellular metabolism as a therapeutic strategy.

## Conclusion and Future Perspectives

Over the past few years, the pivotal role of metabolism in the regulation of EC behavior is becoming increasingly recognized. However, more efforts are needed to fully characterize the metabolic roadmap of ECs in health and disease, requiring global untargeted metabolomics analysis of patient samples in combination with the generation of EC-specific metabolic knockout animals. To date, therapeutic strategies to combat pathological angiogenesis primarily rely on VEGF signaling blockade (59, 60). However, efficacy of these treatments and improvements in survival are limited by acquired refractoriness and drug resistance (59, 60). There is, thus, an unmet need to develop additional anti-angiogenic therapies that operate *via* fundamentally different, complementary mechanisms. The concept of targeting metabolic pathways for improving current therapies is still in its infancy. Initial proof of concept has already been provided in preclinical animal studies, whereby blockade of PFKFB3 proved to be efficacious in reducing pathological angiogenesis of skin and bowel inflammation, and enhancing the anti-angiogenic effects of VEGF inhibitors (24, 56), and promoting tumor vessel normalization with reduced metastasis and improved responses to chemotherapy (19). Of note, in a preclinical mouse model of wet age-related macular degeneration, systemic administration of the PFKFB3 blocker 3PO inhibited choroidal neovascularization (24). Similarly, pathological ocular neovascularization is reduced by systemic administration of etomoxir, a CPT1 blocker (30).

A growing body of evidence suggests that EC functions can be modulated by metabolites in the blood or released by surrounding tissues. An evident question is whether dietary supplementation of metabolites might act in concert with current therapeutic strategies to ease vascular disease symptoms? In support of this concept, supplementation of acetate (a precursor of acetyl-CoA) promotes (lymph)-angiogenesis, *in vitro* as well as *in vivo* (3, 61).

## Author Contributions

AZ, JK, CD, and PC wrote the paper and commented on the manuscript.

## Conflict of Interest Statement

The authors declare that the research was conducted in the absence of any commercial or financial relationships that could be construed as a potential conflict of interest.
